# Impact of heteroresistance on treatment outcomes of people with drug-resistant TB

**DOI:** 10.5588/ijtldopen.24.0343

**Published:** 2024-10-01

**Authors:** R. Crowder, M. Kato-Maeda, B. Schwem, A. dela Tonga, D.M. Geocaniga-Gaviola, E. Lopez, C.L. Valdez, A.R. Lim, N. Hunat, A.G. Sedusta, C.A. Sacopon, G.A.M. Atienza, E. Bulag, D. Lim, J. Bascuña, K. Shah, R.P. Basillio, C.A. Berger, M.C.D.P. Lopez, S. Sen, C. Allender, M. Folkerts, U. Karaoz, E. Brodie, S. Mitarai, A.M.C. Garfin, M.C. Ama, D.M. Engelthaler, A. Cattamanchi, R. Destura

**Affiliations:** ^1^ Division of Pulmonary and Critical Care Medicine, San Francisco General Hospital, University of California San Francisco, San Francisco, CA, USA;; ^2^ Center for Tuberculosis, University of California San Francisco, San Francisco, CA, USA;; ^3^ University of the Philippines, National Institutes of Health, Manila, Philippines;; ^4^ Philippines Department of Health, Manila, Philippines;; ^5^ National Tuberculosis Reference Laboratory, Research Institute for Tropical Medicine, Muntinlupa, Philippines;; ^6^ Department of Preventive Medicine, University of Tennessee Health Science Center, Memphis, TN, USA;; ^7^ Pathogen and Microbiome Division, Translational Genomics Research Institute, Flagstaff, AZ, USA;; ^8^ Ecology Department, Lawrence Berkeley National Laboratory, Berkeley, CA, USA;; ^9^ Research Institute for Tuberculosis, Tokyo, Japan;; ^10^ Division of Pulmonary Diseases and Critical Care Medicine, University of California Irvine, Irvine, CA, USA.

**Keywords:** Drug resistance-associated mutations, DR-TB, tuberculosis

## Abstract

**BACKGROUND:**

Poor treatment outcomes among people with drug-resistant TB (DR-TB) are a major concern. Heteroresistance (presence of susceptible and resistant *Mycobacterium tuberculosis* in the same sample) has been identified in some people with TB, but its impact on treatment outcomes is unknown.

**METHODS:**

We used targeted deep sequencing to identify mutations associated with DR-TB and heteroresistance in culture samples of 624 people with DR-TB. We evaluated the association between heteroresistance and time to unfavorable treatment outcome using Cox proportional hazards regression.

**RESULTS:**

The proportion of drug-resistant isolates with a known mutation conferring resistance was lower for streptomycin (45.2%) and second-line injectables (79.1%) than for fluoroquinolones (86.7%), isoniazid (93.2%) and rifampin (96.5%). Fifty-two (8.3%) had heteroresistance, and it was more common for fluoroquinolones (4.6%) than rifampin (2.2%), second-line injectables (1.4%), streptomycin (1.7%), or isoniazid (1.3%). There was no association between heteroresistance and time to unfavorable outcome among people with multidrug-resistant TB (adjusted hazard ratio [aHR] 1.74, 95% CI 0.39–7.72) or pre-extensively DR-TB (aHR 0.65, 95% CI 0.24–1.72).

**CONCLUSIONS:**

Heteroresistance was relatively common (8.3%) among people with DR-TB in the Philippines. However, we found insufficient evidence to demonstrate an impact on unfavorable treatment outcomes.

## RÉSUMÉ

**CONTEXTE:**

Les résultats médiocres du traitement chez les personnes atteintes de TB résistante aux médicaments (DR-TB, pour l’anglais « *drug-resistant TB* ») constituent une préoccupation significative. L'hétérorésistance, caractérisée par la coexistence de souches sensibles et résistantes de *Mycobacterium tuberculosis* dans un même échantillon, a été observée chez certains patients, mais les conséquences de cette situation sur l'efficacité des traitements demeurent incertaines.

**MÉTHODES:**

Nous avons recouru au séquençage profond ciblé afin d'identifier les mutations liées à la DR-TB et à l'hétérorésistance dans les échantillons de culture provenant de 624 personnes atteintes de DR-TB. Nous avons analysé le lien entre l'hétérorésistance et le délai jusqu'à l'issue défavorable du traitement en utilisant une régression des risques proportionnels de Cox.

**RÉSULTATS:**

La proportion d’isolats, présentant une mutation connue associée à la résistance, était inférieure pour la streptomycine (45,2%) et les médicaments injectables de deuxième ligne (79,1%) par rapport aux fluoroquinolones (86,7%), à l'isoniazide (93,2%) et à la rifampicine (96,5%). Parmi les isolats, cinquante-deux (8,3%) manifestaient une hétérorésistance, plus courante pour les fluoroquinolones (4,6%) que pour la rifampicine (2,2%), les médicaments injectables de deuxième ligne (1,4%), la streptomycine (1,7%) ou l'isoniazide (1,3%). Aucune association n'a été observée entre l'hétérorésistance et un délai d'évolution défavorable chez les patients atteints de TB multirésistante (rapport de risque ajusté [aHR] 1,74 ; IC à 95% 0,39–7,72) ou de DR-TB pré-extensive (aHR 0,65 ; IC à 95% 0,24–1,72).

**CONCLUSIONS:**

L'hétérorésistance a été observée de manière relativement fréquente (8,3%) chez les personnes atteintes de DR-TB aux Philippines. Néanmoins, nous n'avons pas identifié de preuves suffisantes pour établir un lien avec des résultats de traitement défavorables.

Globally, the treatment success rate is 88% for people with drug-susceptible TB (DS-TB) and 63% for people with multidrug-resistant TB (MDR-TB), defined as resistant to at least isoniazid (INH) and rifampin (RIF), or RIF-resistant TB (RR-TB).^1^ The reasons for unfavorable outcomes are multifactorial, including lower efficacy of second-line drugs for drug-resistant (DR) *Mycobacterium tuberculosis* (MTB), immune incompetence, and poor adherence to treatment.^1–4^ More recently, heteroresistance has been associated with poor treatment outcomes.^5–7^

Heteroresistance (defined as the presence of both DR and DS-TB strains in the same sample) has been associated with treatment failure in some studies,^5,6,8^ but not in others.^9,10^ Most published studies used technology that requires at least 5–10% of the bacterial population to harbor the mutation associated with resistance to be detected.^11^ If the proportion of the resistant population falls below this threshold, resistance will be missed by genotypic methods such as Xpert^®^ MTB/RIF Ultra (Cepheid, Sunnyvale, CA, USA) and GenoType™ MTBDR*plus* (Hain Lifescience, Nehren, Germany), hindering the selection of appropriate treatment.^11–13^

We performed targeted deep sequencing, which enables extremely low-level detection of resistant alleles, to assess the frequency of heteroresistance to anti-TB drugs and to determine its impact on treatment outcomes in people with TB at least resistant to INH and RIF in the Philippines, a high DR-TB burden country.^1^ We included heteroresistance to INH and RIF in our analysis because previous studies found that heteroresistance to these drugs in people with MDR-TB was associated with poor outcomes, possibly because of failure to provide optimal treatment for the susceptible MTB subpopulations.^5^

## METHODS

The study was approved by the Institutional Review Boards of the University of California San Francisco, CA, USA; the Research Institute of Tropical Medicine, the Philippines Department of Health, Manila; and the University of the Philippines, Manila, the Philippines.

### Study design and setting

We conducted a retrospective cohort study of people with DR-TB enrolled in the Philippines Programmatic Management of Drug-resistant TB of the National TB Program (NTP) between 2013 and 2016.^14^ The NTP used Xpert MTB/RIF in sputum in individuals presumed to have TB, culture in those with RIF resistance or presumed to have DR-TB and drug susceptibility testing (DST) using critical concentrations recommended at the time of the diagnosis by the WHO for Löwenstein-Jensen (LJ) or BACTEC™ MGIT™ 960 (Becton Dickinson, Sparks, MD, USA), respectively: RIF, 40.0 μg/mL and 1.0 μg/mL; INH, 0.2 μg/mL and 0.1 μg/mL; streptomycin (SM), 4.0 μg/mL and 1.0 μg/mL, levofloxacin, 1 μg/mL (up to 2018) for LJ; kanamycin, 30.0 μg/mL and 2.5 μg/mL; amikacin, 40.0 μg/mL and 1.0 μg/mL; capreomycin, 40.0 μg/mL and 2.5 μg/mL.^15^

### Study population

We included people who, according to programmatic data from the National Tuberculosis Reference Laboratory (NTRL), had pre-extensively drug-resistant (pre-XDR-TB), MDR-TB plus resistance to second-line injectables (SLI), and MDR-TB between 2013 and 2016. The sample size was determined by the budget available to perform single molecule with overlapping reads (SMOR). We, therefore, prioritized people with pre-XDR-TB and only included a random sample of people with MDR-TB. We excluded people with extrapulmonary TB, without treatment information, without viable *M. tuberculosis*, and valid SMOR data. We restricted the analysis to individuals on treatment for over 28 days to reduce bias from delayed presentation.

### Procedures

We collected demographic and clinical details from the Integrated Tuberculosis Information System (ITIS), the electronic case notification system used by the NTP, and standardized treatment cards. For individuals with an initial treatment outcome of ‘Treatment Failed’ or ‘Lost to Follow-up,’ we searched ITIS and NTP records to identify further treatment episodes, called their contact of record, and/or conducted home visits to ascertain the most recent treatment outcome and vital status.

Phenotypic DST results were obtained from the NTRL database. Because of changes in the critical concentrations for fluoroquinolones (FQs), we performed phenotypic DST using moxifloxacin 1.0 μg/mL.^15^

Targeted deep sequencing was performed using SMOR for genes associated with resistance to INH *(inh*A*, kat*G*)*, RIF *(rpo*B*)*, FQs *(gyr*A, *gyr*B*)*, SLI (*eis*, *rrs*, *tlyA*, *gid*B*) and streptomycin* (*rpsL* and *rrs*).^16^ SMOR is a method that identifies heteroresistance with confidence at the level of 0.1% and, therefore, is more suitable than other genotyping methods to identify heteroresistance when the resistant population is lower than 5–10% of the total bacillary population. Amplicons were pulled, and libraries were prepared for Illumina-based sequencing (Illumina, San Diego, CA, USA) to target an average of 50,000x coverage.^17^ Measures to avoid laboratory cross-contamination included tests of batches of 10 samples or less, strict protocols, physical separation of pre- and post-polymerase chain reaction areas, and no-template controls. DNA from a confirmed pan-susceptible MTB H37Ra (American Type Culture Collection 25177) was used as a sequencing error control throughout the SMOR assay.^17^ Samples with low coverage were repeated once.

Sequence data were analyzed using the in-house TB Amplicon Sequencing Analysis Pipeline software^18^ Discordant paired reads from the same DNA molecule were excluded. Validation studies using mixtures of DR- and DS-TB demonstrated that at least five mutant alleles of 5,000 reads were required for robust identification of resistant sub-populations at 0.1% or more.^17^

### Definitions

At the time of the study population selection, pre-XDR was defined as MDR-TB with additional resistance to SLI or FQs and XDR as MDR-TB with additional resistance to SLI and FQs.^19^ However, for the analysis, we used the 2021 WHO definitions that define pre-XDR as MDR-TB with additional resistance to FQs.^20^ We included those with MDR-TB plus SLI resistance (SLIR) as a separate group.

We included mutations associated with resistance based on the recently published WHO catalog of mutations.^16^ We classified heteroresistance based on the proportion of the bacterial population with a drug resistance-associated allele: rare for 0.1 to 0.99%, micro for 1 to 10%, and macro for >10% to <95%.

Unfavorable treatment outcomes were defined as death or treatment failure. The date of unfavorable outcome was recorded as the date of death or the date of most recent treatment failure. Those who were cured/treatment completed or lost to follow-up (LTFU) were censored on the date of last medication dosing. We included up to 730 days of follow-up in the analysis.

For people missing height, body mass index (BMI) was imputed using weight and median height by sex among those not missing height. If missing, the highest pre-treatment sputum smear grade was used instead of the baseline smear.

### Statistical analysis

The analysis was stratified by drug resistance type (MDR-TB, MDR-TB plus SLIR, and pre-XDR-TB). The unadjusted association between heteroresistance and time to unfavorable treatment outcome was assessed using log-rank tests. The adjusted association was modeled using Cox proportional hazards regression, controlling for known risk factors for poor treatment outcome (age, sex, BMI, cavitary chest X-ray, sputum smear grade at baseline). Other characteristics were adjusted for only if associated with unfavorable outcomes (*P* < 0.2) in bivariate analysis.

## RESULTS

### Study population

**Figure 1.**
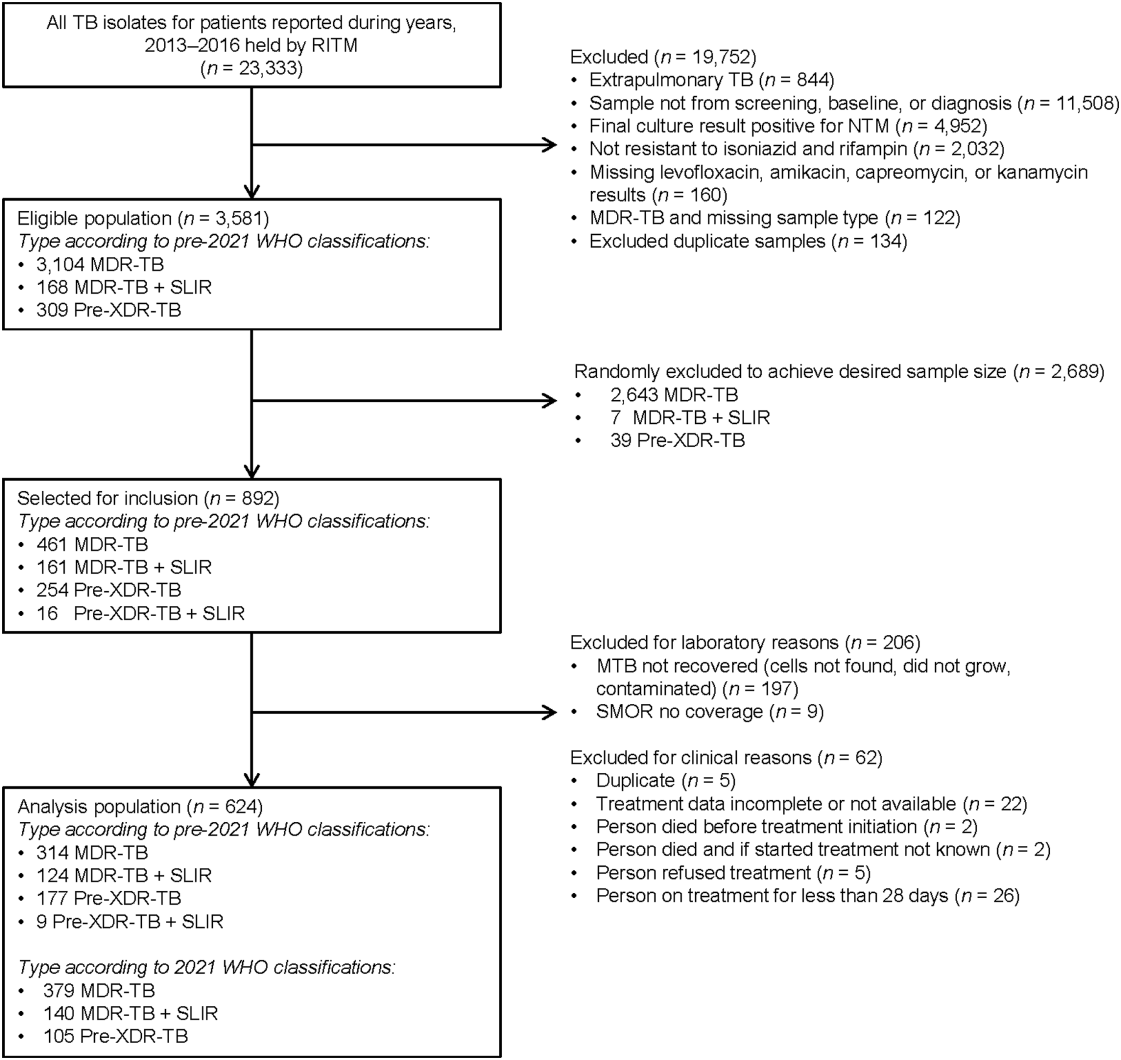
Study population selection and description of exclusion criteria. RITM = Research Institute for Tropical Medicine; NTM = nontuberculous mycobacteria; MDR-TB = multidrug-resistant TB; SLIR = second-line injectable resistant; XDR-TB = extensively drug-resistant; SMOR = single molecule overlapping reads.

The eligible population included 3,104 people with MDR-TB, 168 with MDR-TB plus SLIR-TB and 309 with pre-XDR-TB (Figure 1). We randomly excluded 2,689 people, leaving 461 people with MDR-TB and 431 with MDR plus SLIR or pre-XDR-TB. We excluded 206 people for laboratory reasons and 62 for clinical reasons. Thus, 624 people with DR-TB were included in this analysis: 379 with MDR-TB, 140 with MDR plus SLIR-TB, and 105 with pre-XDR-TB. Among the 624 people with DR-TB, 304 had isolates resistant to SM.

### Frequency of mutation

The distributions of mutations associated with drug resistance are shown in the Supplementary Tables S1 and S2. Most isolates with resistance to INH (573/615, 93.2%) and RIF (602/624, 96.5%) had mutations associated with resistance. Among those with INH resistance, 360/615 (58.5%) had the *kat*G S315T mutation, and an additional 6.0% (*n* = 37) had *kat*G S315T in combination with inhAc-15t. Among the 602 isolates with mutations associated with RIF resistance, 576 (95.7%) had a single mutation, and 26 (4.3%) had more than one mutation, totaling 632 mutations. The most frequent mutation was *rpo*B S531L alone (*n* = 375) or with other mutations (*n* = 17) and accounted for 62% of the mutations identified, followed by mutations in the *rpo*B codon 526 (H526Y: 64/632, 10.1%; H526D: 29/632, 4.6%; and H526R: 27/632, 4.3%).

Among the 105 FQ-resistant isolates, 91 (86.7%) had mutations associated with resistance in *gyr*A, 70 (76.9%) had a single mutation, and 21 (23%) had between 2 and 7 mutations, totaling 128 mutations. The remaining isolates (*n* = 14, 13.3%) did not have known resistance-conferring mutations. Among the 128 mutations, the most frequent were gD94G (*n* = 44, 34.4%), A90V (*n* = 38, 29.7%) and D94A (*n* = 13, 10.2%). Among the 502 isolates phenotypically susceptible to FQ, two had the mutation A90V, and one had the mutation D94G. Data from 16 isolates were excluded because of incomplete moxifloxacin data. Of the 134 isolates with SLIR, 106 (79.1%) had a *1401G* mutation. The remaining 28 (20.9%) had no known resistance-conferring mutations. Of the 490 isolates phenotypically susceptible to SLI, 6 (1.2%) had drug resistance-associated mutations. Among 304 isolates phenotypically resistant to SM, 11 had low SMOR coverage. Only 132 of 293 isolates (45.2%) had known mutations associated with SM resistance, the most frequent being A514C (*n* = 123, 93.2%). Among the 320 SM-susceptible isolates, 19 had low SMOR coverage, and nine isolates of the 301 with SMOR coverage (3.0%) had drug resistance-associated mutations.

### Heteroresistance

Among the 624 people with DR-TB, 52 (8.3%) had MTB cultures with heteroresistance to at least one drug. When comparing the characteristics of people with and without heteroresistance, we found that those with heteroresistance were older in the MDR-TB and pre-XDR-TB groups and less likely to have previous treatment with first-line drugs in the MDR-TB and MDR + SLIR groups (Table 1).

**Table 1.** Clinical and demographic characteristics of 624 patients included in the study, 2013–2016.

	People treated for MDR-TB (*n* = 379)		People treated for MDR-TB + SLIR TB (*n* = 140)		People treated for pre-XDR-TB (*n* = 105)	
	HR (*n* = 13) *n* (%)	No HR (*n* = 366) *n* (%)	HR (*n* = 8) *n* (%)	No HR (*n* = 132) *n* (%)	HR (*n* = 31) *n* (%)	No HR (*n* = 74) *n* (%)
Age, years, median [IQR]	52 [42–56]	43 [33–52]	34.5 [27–46]	34 [26–45]	49 [34–58]	35 [24–54]
Female	5 (38.5)	116 (31.7)	0 (0.0)	32 (24.2)	11 (35.5)	25 (33.8)
Body mass index, kg/m^2^, median [IQR]	19 [16–20]	18 [16–21]	18 [17–20]	18 [16–21]	18 [15–19]	18 [15–22]
National Capital Region	3 (23.1)	109 (29.8)	7 (87.5)	95 (72.0)	10 (32.3)	26 (35.1)
Cavitary CXR	7 (53.9)	115 (31.4)	0 (0.0)	18 (13.6)	4 (12.9)	20 (27.0)
Previous TB treatment	11 (84.6)	354 (96.7)	4 (50.0)	113 (85.6)	30 (96.8)	72 (97.3)
Heteroresistance						
Macro (>10%)	6 (46.2)	—	5 (62.5)	—	21 (67.7)	—
Micro (≤10%)	2 (15.4)	—	1 (12.5)	—	7 (22.6)	—
Rare	5 (38.5)	—	2 (25.0)	—	3 (9.7)	—
>1 resistant population	2 (25.0)	3 (2.3)	2 (25.0)	3 (2.3)	3 (9.7)	2 (2.7)

MDR-TB = multidrug-resistant TB; SLIR = second-line injectable-resistant; XDR-TB = extensively drug-resistant TB; HR = heteroresistance; IQR = interquartile range; CXR = chest X-ray.

Of 3,064 valid heteroresistance results available (*n* = 615 for INH, *n* = 624 for RIF, *n* = 624 for SLI, *n* = 594 for SM, and *n* = 607 for FQs), 69 (2.3%) showed heteroresistance. The proportion with heteroresistance differed by drug class (*P* < 0.001), with FQs (*n* = 28/607, 4.6%) having a relatively higher proportion when compared to the other drugs (Table 2).

**Table 2.** Frequency of heteroresistance and phenotypic resistance by anti-TB drug (3,064 drugs evaluated in 624 isolates).*

	Total			Susceptible			Resistant		
Drugs/target	Heteroresistance *n/N* (%)	Macro *n* (%)	Micro/rare *n* (%)	Heteroresistance *n/N* (%)	Macro *n* (%)	Micro/rare *n* (%)	Heteroresistance *n/N* (%)	Macro *n* (%)	Micro/rare *n* (%)
Isoniazid	8/615 (1.3)	6 (1.0)	2 (0.3)	—	—	—	8/615 (1.3)	6 (1.0)	2 (0.3)
Rifampicin	14/624 (2.2)	9 (1.4)	5 (0.8)	—	—	—	14/624 (2.2)	9 (1.4)	5 (0.8)
Second-line injectables	9/624 (1.4)	6 (1.0)	3 (0.5)	3/490 (0.6)	3 (0.6)	0 (0.0)	6/134 (4.4)	3 (2.2)	3 (2.2)
Streptomycin	10/594 (1.7)	5 (0.8)	5 (0.8)	4/301 (1.3)	1 (0.3)	3 (1.0)	6/293 (2.0)	4 (1.4)	2 (0.7)
Fluoroquinolones	28/607 (4.6)	17 (2.8)	11 (1.8)	2/502 (0.4)	0 (0.0)	2 (0.4)	26/105 (24.8)	17 (16.2)	9 (8.6)
Total	69/3,064 (2.3)	43 (1.4)	26 (0.8)	9/1293 (0.7)	4 (0.3)	5 (0.4)	60/1,771 (3.4)	39 (2.2)	21 (1.2)

* All *P*-values <0.001 for association between heteroresistance and phenotypic resistance by drug class.

Heteroresistance was typical to one drug class (43/52, 82.7%) but was present for two or more drugs in nine people (to two drugs in four people, to three drugs in three people, and four drugs in two people). Among the 69 events of heteroresistance, rare-, micro-, and macro-heteroresistance were observed in respectively 13 (18.8%), 13 (18.8%), and 43 (62.3%) (Supplementary Table S6, Supplementary Figure S1).

The proportion of isolates with heteroresistance to individual drugs was higher when the corresponding phenotypic result was resistant (60/1,771, 3.4% vs. 9/1,293, 0.7%; *P* < 0.0001). The frequency of heteroresistance for FQs was especially higher among phenotypically FQ-resistant MTB (24.8%, *P* = 0.00011 in comparison with other drugs in DR MTB) (Table 2).

Among the nine events of heteroresistance in phenotypically susceptible isolates, 4 were macro-heteroresistance, and 5 were micro/rare-heteroresistance (Table 2).

### Impact on treatment outcome

Among 624 people with DR-TB, the original treatment outcome recorded was cured for 288 (46.2%), treatment completed for 74 (11.9%), treatment failed for 21 (3.4%), died for 51 (8.2%), LTFU for 189 (30.3%), and still on treatment for one at the last time of data collection, over 2 years after treatment initiation. After tracing the 210 who had an outcome of treatment failed or LTFU, 24 were re-categorized as having a favorable outcome (cured or treatment completed); 62 were categorized as having an unfavorable outcome (54 died, 8 failed treatment), and 124 retained the outcome of LTFU. Thus, after tracing results were incorporated, 386 (61.9%) had a favorable outcome, 113 (18.1%) had an unfavorable outcome, 124 (19.9%) were LTFU, and one remained on treatment >2 years after treatment initiation.

**Figure 2.**
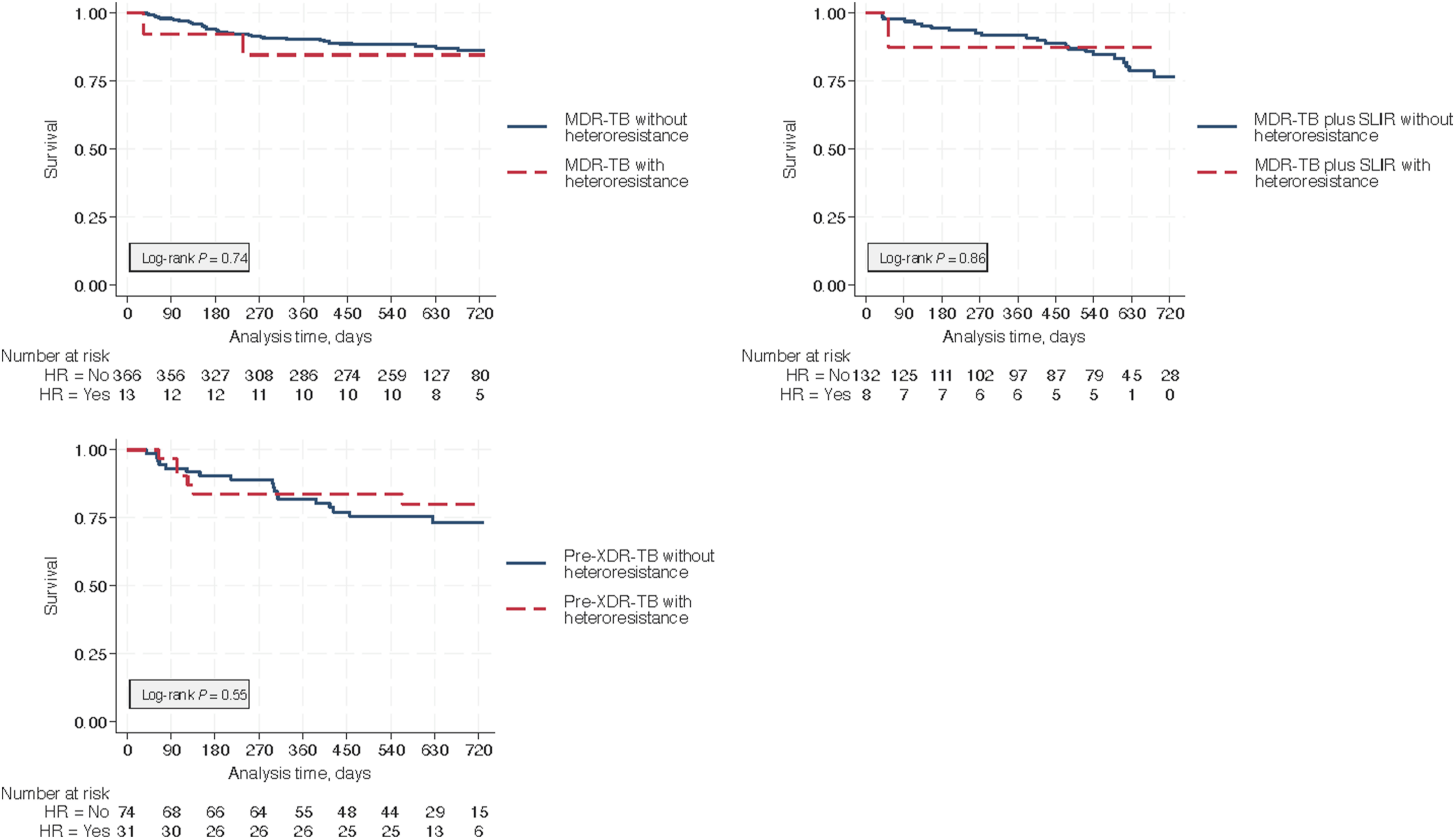
Kaplan–Meier curves showing the time-to-unfavorable outcome by drug resistance classification. Time-to-unfavorable outcomes (death or treatment failure) was not associated with heteroresistance among patients with MDR-TB (top left, *n* = 379), MDR- plus SLIR-TB (top right panel, *n* = 140), or pre-XDR-TB (bottom left, *n* = 105). MDR-TB = multidrug-resistant TB; HR = heteroresistance; SLIR = second-line injectable resistant; XDR-TB = extensively drug-resistant TB.

We found insufficient evidence of an association between heteroresistance and time to unfavorable treatment outcome for people with MDR-TB, MDR plus SLIR-TB, or pre-XDR-TB up to 730 days after treatment initiation (see Figure 2: Kaplan-Meier curves). After adjusting for known demographic and clinical risk factors, heteroresistance was not associated with unfavorable outcomes in people with MDR-TB (adjusted hazard ratio [aHR] 1.74, 95% confidence interval [CI] 0.39–7.72; *P* = 0.47), MDR- plus SLIR-TB (aHR 1.13, 95% CI 0.14–9.14; *P* = 0.91) or pre-XDR-TB (aHR 0.65, 95% CI 0.24–1.72; *P* = 0.38) (Table 3). Imputing chest X-rays using sex, age, BMI, sputum smear grade, region of the Philippines, and year of treatment did not meaningfully change the estimates (Supplementary Table S7). The wide CIs and visual impression from the Kaplan–Meier curves indicate substantial uncertainty in the estimates.

**Table 3.** Hazard of unfavorable outcome associated with heteroresistance.

Heteroresistance	*n*	HR (95% CI)	aHR (95% CI)*	*P*-value*
MDR-TB	379	1.27 (0.31–5.24)	1.74 (0.39–7.72)^†^	0.47
MDR-TB plus SLIR	140	0.84 (0.11–6.24)	1.13 (0.14–9.14)^†^	0.91
Pre-XDR-TB	105	0.76 (0.30–1.90)	0.65 (0.24–1.72)	0.38

* Adjusted for known risk factors for poor treatment outcome (age, sex, body mass index, cavitary chest X-ray, sputum smear grade at baseline). ^†^Also adjusted for the region of the Philippines (national capital region vs. other).

HR = hazard ratio; CI = confidence interval; aHR = adjusted HR; MDR-TB = multidrug-resistant TB; SLIR = second-line injectable resistant; XDR-TB = extensively drug-resistant TB.

## DISCUSSION

In this population-based study of people with DR-TB in the Philippines, nearly all INH and RIF-resistant isolates had known mutations, while only 45.1%, 79.1%, and 86.7% of the isolates resistant to SM, SLI, and FQ, respectively, had known mutations. When using a highly sensitive technique such as SMOR, heteroresistance was relatively common (8.3%) but contributed to discordance between the phenotypic and genotypic susceptibility in less than 1% of our study population. Interestingly, all six cases had micro or macro-heteroresistance and, therefore, should have been detected by the phenotypic methods. The frequency of heteroresistance found in this study cannot be directly compared against published studies because of the differing study populations and methods used to identify heteroresistance.^6,8,11,21–26^

In the Philippines, the presence of heteroresistance was not associated with unfavorable treatment outcomes, which is in line with previous smaller studies.^9,10^ However, a prior study in Botswana, in which 69% of the study population had HIV co-infection and MDR-TB, found that heteroresistance to INH or RIF was associated with unfavorable outcomes. A second study that showed an association between heteroresistance and unfavorable outcomes used MTB genotyping and culture to define heteroresistance. Neither of these studies adjusted for possible confounders.^5,8^ Although heteroresistance is the first step for a susceptible strain to become resistant,^7^ it is likely that current molecular methods are sufficient to identify clinically relevant levels of small populations of drug-resistant TB,^11^ thus allowing for appropriate treatment and decreasing the clinical impact of having heteroresistance. In this population-based study of people with DR-TB, a key finding was that 13.3% of phenotypically FQ-resistant MTB isolates lacked known mutations associated with resistance. Therefore, one in eight people with FQ-resistant TB in the Philippines would not be detected with the current molecular tests and would likely be treated with suboptimal drug regimens.^27,28^

Key strengths of our study include the population-based sample of people with DR-TB, the use of targeted deep sequencing, and adjustment for other factors that impact treatment outcomes. The limitations of our study include the use of subcultures from the original culture, which may not represent the bacterial population in the lungs. Programmatic clinical data were sometimes incomplete, limiting our ability to entirely control for factors associated with treatment outcomes. Last, we were underpowered to assess whether different levels of heteroresistance and heteroresistance to individual drugs/drug classes are associated with unfavorable treatment outcomes.

In conclusion, we found that 8.3% of the people in the Philippines with DR-TB had heteroresistance. We found insufficient evidence to demonstrate an impact on unfavorable outcomes. We also found that 13.3% of FQ-resistant isolates did not have mutations known to be associated with resistance, which will hinder the exclusive use of molecular methods to identify fluoroquinolone resistance.

## Supplementary Material


