# Supplementary material for: Impact of heteroresistance on treatment outcomes of people with drug-resistant TB
Source: IJTLD Open. 2024 Oct 1;1(10):466–72. doi: 10.5588/ijtldopen.24.0343 (PMC11467852; doi:10.5588/ijtldopen.24.0343)
Supplement: Supplementary file 1 [file ijtldopen24-0343_SupplementalData1.pdf]

http://dx.doi.org/10.5588/ijtldopen.24.0343

Impact of heteroresistance on treatment outcomes of people with drug-resistant TB

Supplementary Data

Table S1. Mutation pattern in each of 624 MTB isolates 2

Table S2. Frequency of mutations per drug class 4

Table S3. Clinical and demographic characteristics of 379 patients treated for MDR TB in the Philippines, 2013-2016 6

Table S4. Clinical and demographic characteristics of 140 patients treated for MDR plus SLIR TB in the Philippines, 2013-2016 7

Table S5. Clinical and demographic characteristics of 105 patients treated for Pre-XDR TB in the Philippines, 2013-2016 8

Table S6. Distribution of degree of heteroresistance based on the phenotypic susceptibility test 9

Figure S1. Frequency of heteroresistance by drug class/target 9

Table S7. Hazard of unfavorable outcome associated with heteroresistance in multiple imputation analysis 10

Table S1. Mutation pattern in each of 624 MTB isolates

Mutation	Resistant n (%)	Susceptible n (%)
**Isoniazid** (615 resistant MTB isolates with valid data)		
**katGS315T**	360 (58.5)	
**inhAc-15t**	159 (25.9)	
**inhAc-15t, katGS315T**	37 (6.0)	
**katGS315N**	16 (2.6)	
**inhA KS315N**	1 (0.2)	
**No mutation**	42 (6.8)	
**Rifampicin** (624 resistant MTB isolates), All mutations in the *rpoB* gene		
**S531L**	375 (60.1)	
**S531L H526Y**	3 (0.5)	
**S531L S531F**	2 (0.3)	
**S531L H526L**	2 (0.3)	
**S531L H526D**	2 (0.3)	
**S531L D516V**	2 (0.3)	
**S531L S531W**	1 (0.2)	
**S531L S522Q**	1 (0.2)	
**S531L S522L**	1 (0.2)	
**S531L L533P**	1 (0.2)	
**S531L H526Y D516Y L533P**	1 (0.2)	
**S531L H526R L533P**	1 (0.2)	
**H526Y**	58 (9.3)	
**H526Y H526C H526R**	1 (0.2)	
**H526Y H526R**	1 (0.2)	
**H526R**	23 (3.7)	
**H526R L533P**	1 (0.2)	
**H526D**	23 (3.7)	
**H526D Q513K**	4 (0.6)	
**S531W**	19 (3.0)	
**D516V**	19 (3.0)	
**H526C**	14 (2.2)	
**L533P**	11 (1.8)	
**H526L**	8 (1.3)	
**H526L D516V**	1 (0.2)	
**D516Y**	8 (1.3)	
**S522L**	8 (1.3)	
**Q513P**	3 (0.5)	

**D516F**	3 (0.5)	
**Q513L**	2 (0.3)	
**S531F**	1 (0.2)	
**S531F D516V**	1 (0.2)	
**Q513K**	1 (0.2)	
**No mutation**	22 (3.5)	
**Fluoroquinolones** (105 resistant, 502 susceptible MTB isolates with valid data). All mutations in the *gyrA* gene.		
**D94G**	26 (24.8)	1 (0.2)
**D94G A90V**	3 (2.9)	
**D94G A90V D94N**	3 (2.9)	
**D94G A90V D94A**	2 (1.9)	
**D94G A90V D94H**	2 (1.9)	
**D94G D94A**	2 (1.9)	
**D94G D94H D94Y**	1 (1.0)	
**D94G D94N**	1 (1.0)	
**D94G A90V S91P D94A D94H D94N D94Y**	1 (1.0)	
**D94G A90V S91P**	1 (1.0)	
**D94G G88C A90V S91P**	1 (1.0)	
**D94G S91P**	1 (1.0)	
**A90V**	22 (21.0)	2 (0.4)
**A90V S91P**	2 (1.9)	
**A90V D94A**	1 (1.0)	
**D94A**	7 (6.7)	
**D94N**	7 (6.7)	
**D94Y**	5 (4.8)	
**D94H**	2 (1.9)	
**S91P**	1 (1.0)	
**No mutation**	14 (13.3)	499 (99.4)
**Second-line Injectables** (134 resistant, 490 susceptible MTB isolates with valid data). All mutations in the *rrs* gene.		
**1401G**	106 (79.1)	5 (1.0)
**1484T**	0 (0.0)	1 (0.2)
**No mutation**	28 (20.9)	484 (98.8)
**Streptomycin** (293 resistant, 301 susceptible MTB isolates with valid data). All mutations in the *rrs* gene.		
**A514C**	123 (42.1)	7 (2.3)
**C517T**	9 (3.1)	2 (0.7)
**No mutation**	161 (54.9)	292 (97.0)

Table S2. Frequency of mutations per drug class

Mutation	Resistant n (%)	Susceptible n (%)
**Isoniazid** (611 mutations among INH resistant MTB)		
**katGS315T**	397 (65.0)	
**inhAc-15t**	197 (32.2)	
**katGS315N**	17 (2.8)	
**Rifampicin** (632 mutations among RIF resistant MTB, 26 isolates had more than one mutation)		
**S531L**	392 (62.0)	
**H526Y**	64 (10.1)	
**H526D**	29 (4.6)	
**H526R**	27 (4.3)	
**D516V**	23 (3.6)	
**S531W**	20 (3.2)	
**H526C**	15 (2.4)	
**L533P**	15 (2.4)	
**H526L**	11 (1.7)	
**D516Y**	9 (1.4)	
**S522L**	9 (1.4)	
**Q513K**	5 (0.8)	
**S531F**	4 (0.6)	
**D516F**	3 (0.5)	
**Q513P**	3 (0.5)	
**Q513L**	2 (0.3)	
**S522Q**	1 (0.2)	
**Fluoroquinolones** (128 mutations among FQ resistant and 3 among FQ susceptible MTB)		
**D94G**	44 (34.4)	1 (33.3)
**A90V**	38 (29.7)	2 (66.6)
**D94A**	13 (10.2)	
**D94N**	12 (9.4)	
**D94Y**	7 (5.5)	
**S91P**	7 (5.5)	
**D94H**	6 (4.7)	
**G88C**	1 (0.8)	
**Second-line Injectables** (106 mutations among SLI resistant and 6 among SLI susceptible MTB)		
**1401G**	106 (100.0)	5 (83.3)
**1484T**	0 (0.0)	1 (16.7)

**Streptomycin** (132 mutations among Streptomycin resistant and 9 among Streptomycin susceptible MTB)		
**A514C**	123 (93.2)	7 (77.8)
**C517T**	9 (6.8)	2 (22.2)

MTB: Mycobacterium tuberculosis

Table S3. Clinical and demographic characteristics of 379 patients treated for MDR TB in the Philippines, 2013-2016

	Total, N (%) N=379	Heteroresistance, n (%) n = 13	No heteroresistance, n (%) n = 366	p-value*
**Clinical and demographic characteristics**				
Age, median (IQR)	43 (33-52)	52 (42-56)	43 (33-52)	0.06
Female	121 (31.9)	5 (38.5)	116 (31.7)	0.61
Body mass index				
Normal	129 (34.0)	7 (53.9)	122 (33.3)	
Underweight	200 (52.7)	5 (38.5)	195 (53.3)	0.48
Overweight	43 (11.4)	1 (7.7)	42 (11.5)	
Obese	7 (1.9)	0 (0.0)	7 (1.9)	
National Capital Region	112 (29.5)	3 (23.1)	109 (29.8)	0.60
Baseline CXR				
Cavitary	141 (37.2)	7 (53.9)	115 (31.4)	0.23
Non-cavitary	122 (32.2)	3 (23.1)	138 (37.7)	
Unknown	116 (30.6)	3 (23.1)	113 (30.9)	
Smear grade^				
Negative or scanty	94 (24.8)	2 (15.4)	92 (25.1)	
(1+)	108 (28.5)	4 (30.8)	104 (28.4)	0.87
(2+)	84 (22.2)	3 (23.1)	81 (22.1)	
(3+)	93 (24.5)	4 (30.8)	89 (24.3)	
Previous TB treatment	365 (96.3)	11 (84.6)	354 (96.7)	0.02
**Heteroresistance**				
Macro (>10%)	6 (1.6)	6 (46.2)	-	
Micro (≤10%)	2 (0.5)	2 (15.4)	-	
Rare	5 (1.3)	5 (38.5)	-	
Heteroresistance to:				
Fluoroquinolones	2 (0.5)	2 (15.4)	-	
Second-line injectables	1 (0.3)	1 (7.7)	-	
Isoniazid	2 (0.5)	2 (15.4)	-	
Rifampicin	7 (1.9)	7 (53.9)	-	
Streptomycin	2 (0.5)	2 (15.4)	-	
>1 resistant population	16 (4.2)	2 (15.4)	14 (3.8)	0.04

MDR: Multi-drug resistant, TB: tuberculosis, IQR: interquartile range, CXR: Chest x-ray
*p-value for the association of clinical factor with heteroresistance, using chi-squared test for categorical variables and the Wilcoxon rank-sum to compare median age
^Highest pre-treatment smear grade

Table S4. Clinical and demographic characteristics of 140 patients treated for MDR plus SLIR TB in the Philippines, 2013-2016

	Total, n (%) N = 140	Heteroresistance, n (%) n = 8	No heteroresistance, n (%) n = 132	p-value*
**Clinical and demographic characteristics**				
Age, median (IQR)	34 (26-44.5)	34.5 (27-45.5)	34 (26-44.5)	0.94
Female	32 (22.9)	0 (0.0)	32 (24.2)	0.11
Body mass index				
Normal	52 (37.1)	4 (50.0)	48 (36.4)	
Underweight	77 (55.0)	4 (50.0)	73 (55.3)	0.78
Overweight	10 (7.1)	0 (0.0)	10 (7.6)	
Obese	1 (0.7)	0 (0.0)	1 (0.8)	
National Capital Region	102 (72.9)	7 (87.5)	95 (72.0)	0.34
Baseline CXR				
Cavitary	18 (12.9)	0 (0.0)	18 (13.6)	
Non-cavitary	36 (25.7)	1 (12.5)	35 (26.5)	0.27
Unknown	86 (61.4)	7 (87.5)	79 (59.9)	
Smear grade^				
Negative or scanty	54 (38.6)	3 (37.5)	51 (38.6)	
(1+)	33 (23.6)	4 (50.0)	29 (22.0)	
(2+)	25 (17.9)	0 (0.0)	25 (18.9)	0.37
(3+)	27 (19.3)	1 (12.5)	26 (19.7)	
Missing	1 (0.7)	0 (0.0)	1 (0.8)	
Previous TB treatment	117 (83.6)	4 (50.0)	113 (85.6)	0.008
**Heteroresistance**				
Macro (>10%)	5 (3.6)	5 (62.5)	-	
Micro (≤10%)	1 (0.7)	1 (12.5)	-	
Rare	2 (1.4)	2 (25.0)	-	
Heteroresistance to:				
Fluoroquinolones	0 (0.0)	0 (0.0)	-	
Second-line injectables	6 (4.3)	6 (75.0)	-	
Isoniazid	4 (2.9)	4 (50.0)	-	
Rifampicin	6 (4.3)	6 (75.0)	-	
Streptomycin	4 (2.9)	4 (50.0)	-	
>1 resistant population	5 (3.6)	2 (25.0)	3 (2.3)	0.001

SLIR: Second line injectables resistant, TB: Tuberculosis, CXR: Chest x-ray, IQR: interquartile range

*p-value for the association of clinical factor with heteroresistance, using chi-squared test for categorical variables and the Wilcoxon rank-sum to compare median age

^Highest pre-treatment smear grade

Table S5. Clinical and demographic characteristics of 105 patients treated for Pre-XDR TB in the Philippines, 2013-2016

	Total, n (%) N = 105	Heteroresistance, n (%) n = 31	No heteroresistance, n (%) n = 74	p-value*
**Clinical and demographic characteristics**				
Age, median (IQR)	39 (28-54)	49 (34-58)	35 (24-54)	0.01
Female	36 (34.3)	11 (35.5)	25 (33.8)	0.87
Body mass index				
Normal	31 (29.5)	7 (22.6)	24 (32.4)	
Underweight	61 (58.1)	22 (71.0)	39 (52.7)	0.28
Overweight	10 (9.5)	1 (3.2)	9 (12.2)	
Obese	3 (2.9)	1 (3.2)	2 (2.7)	
National Capital Region	36 (34.3)	10 (32.3)	26 (35.1)	0.78
Baseline CXR				
Cavitary	24 (22.9)	4 (12.9)	20 (27.0)	0.15
Non-cavitary	47 (44.8)	18 (58.1)	29 (39.2)	
Unknown	34 (32.4)	9 (29.0)	25 (33.8)	
Smear grade^				
Negative or scanty	24 (22.9)	4 (12.9)	20 (27.0)	
(1+)	23 (21.9)	6 (19.4)	17 (23.0)	0.07
(2+)	19 (18.1)	10 (32.3)	9 (12.2)	
(3+)	39 (37.1)	11 (35.5)	28 (37.8)	
Previous TB treatment	102 (97.1)	30 (96.8)	72 (97.3)	0.88
**Heteroresistance**				
Macro (>10%)	21 (20.0)	21 (67.7)	-	
Micro (≤10%)	7 (6.7)	7 (22.6)	-	
Rare	3 (2.9)	3 (9.7)	-	
Heteroresistance to:				
Fluoroquinolones	26 (24.8)	26 (83.9)	-	
Second-line injectables	2 (1.9)	2 (6.5)	-	
Isoniazid	2 (1.9)	2 (6.5)	-	
Rifampicin	1 (1.0)	1 (3.2)	-	
Streptomycin	4 (3.8)	4 (12.9)	-	
>1 resistant population	5 (4.8)	3 (9.7)	2 (2.7)	0.13

XDR: Extensively drug-resistant, TB: Tuberculosis, CXR: Chest x-ray, IQR: interquartile range

*p-value for the association of clinical factor with heteroresistance, using chi-squared test for categorical variables and the Wilcoxon rank-sum to compare median age

^Highest pre-treatment smear grade

Table S6. Distribution of degree of heteroresistance based on the phenotypic susceptibility test

	Total of each degree of heteroresistance	Phenotypic resistance n (%)	Phenotypic susceptibility n (%)
**Macro**	43	39 (65.0)	4 (44.4)
**Micro**	13	11 (18.3)	2 (22.2)
**Rare**	13	10 (16.7)	3 (33.3)
**Total**	69	60	9

Figure S1. Frequency of heteroresistance by drug class/target

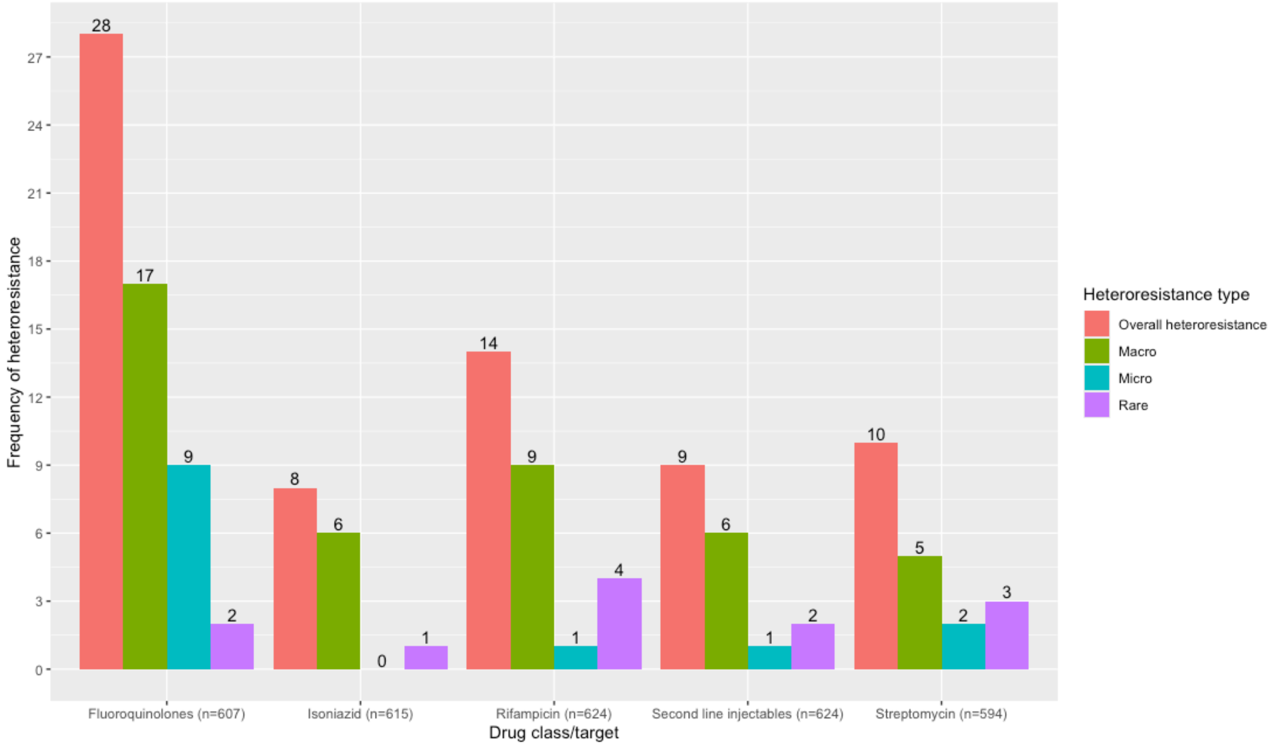

Table S7. Hazard of unfavorable outcome associated with heteroresistance in multiple imputation analysis

	N	Unadjusted Hazard Ratio (95% CI)	Adjusted Hazard Ratio* (95% CI)	P-value*
**Heteroresistance**				
**MDR TB**	379	1.26 (0.31, 5.23)	1.56 (.35, 6.88)	0.59
**MDR plus SLIR**	140	0.83 (0.11, 6.24)	1.14 (0.13, 9.87)	0.90
**Pre-XDR**	105	0.74 (0.29, 1.87)	0.64 (0.24, 1.74)	0.39

* Adjusted for known demographic and clinical risk factors with significance p=0.2 (age, region of the Philippines, cavitary chest X-ray, treatment history, body mass index).

MDR: multi-drug resistant; SLIR: second-line injectable resistant; XDR: extensively drug resistant.
